# RNase H-assisted RNA-primed rolling circle amplification for targeted RNA sequence detection

**DOI:** 10.1038/s41598-018-26132-x

**Published:** 2018-05-17

**Authors:** Hirokazu Takahashi, Masahiko Ohkawachi, Kyohei Horio, Toshiro Kobori, Tsunehiro Aki, Yukihiko Matsumura, Yutaka Nakashimada, Yoshiko Okamura

**Affiliations:** 1 Graduate School of Advanced Sciences of Matter, Hiroshima University, Higashihiroshima, Hiroshima, 739-8530 Japan; 2 Core Research for Evolutional Science and Technology (CREST), Japan Science and Technology Agency (JST), Sanbancho 5, Chiyoda-ku, Tokyo, 102-0075 Japan; 3 Division of Food Biotechnology, Food Research Institute, National Agriculture and Food Research Organization, Tsukuba, Ibaraki 305-8642 Japan; 4 Division of Energy and Environmental Engineering, Hiroshima University, Higashi-Hiroshima, Hiroshima, 739-8527 Japan

## Abstract

RNA-primed rolling circle amplification (RPRCA) is a useful laboratory method for RNA detection; however, the detection of RNA is limited by the lack of information on 3′-terminal sequences. We uncovered that conventional RPRCA using pre-circularized probes could potentially detect the internal sequence of target RNA molecules in combination with RNase H. However, the specificity for mRNA detection was low, presumably due to non-specific hybridization of non-target RNA with the circular probe. To overcome this technical problem, we developed a method for detecting a sequence of interest in target RNA molecules via RNase H-assisted RPRCA using padlocked probes. When padlock probes are hybridized to the target RNA molecule, they are converted to the circular form by SplintR ligase. Subsequently, RNase H creates nick sites only in the hybridized RNA sequence, and single-stranded DNA is finally synthesized from the nick site by phi29 DNA polymerase. This method could specifically detect at least 10 fmol of the target RNA molecule without reverse transcription. Moreover, this method detected GFP mRNA present in 10 ng of total RNA isolated from *Escherichia coli* without background DNA amplification. Therefore, this method can potentially detect almost all types of RNA molecules without reverse transcription and reveal full-length sequence information.

## Introduction

Many recent studies have focused on rolling circle amplification (RCA) for detecting biological molecules^1,2^. We previously reported RNA-primed rolling circle amplification (RPRCA) using phi29 DNA polymerase, circularized probes, and SYBR Green II for the real-time detection of mRNA from living microbes^3^. RPRCA is a simple and easy method, as it does not require reverse transcription (RT), DNase treatment of RNA samples, or a thermal cycler, and it does not entail non-specific DNA amplification.

However, there are two technical disadvantages associated with conventional RPRCA. First, although conventional RPRCA requires the 3′-terminal sequence of target RNA as a primer for DNA synthesis, available sequence database information on the 3′-terminal sequences of prokaryotic mRNAs is limited at present. Currently prevailing RNA-seq technology using next-generation sequencing (NGS) allows the rapid accumulation of growing numbers of prokaryotic mRNA sequences; however, cDNA is usually prepared by RT with random primers in prokaryotic mRNA analysis, and thus, the RNA-seq data lack 3′-terminal sequences. The 3′-terminal sequence of the target mRNA is predictable based on current genomic sequence data. However, available microbial full genome sequences are also limited, specifically regarding metagenomics, as metagenomic sequence data often exist as fragmented information, even when using current NGS and bioinformatics technology.

Second, conventional RPRCA is rarely applied to eukaryotic mRNA detection due to poly(A) tails at 3′ termini, which are approximately 250 bases long^4^. Without RT, the main targets of current RPRCA are certain RNAs that do not contain poly(A) tails such as microRNAs^5^ in eukaryotes. For the simple detection of most types of RNA molecules in prokaryotes and eukaryotes, it is necessary to modify the RPRCA procedure to work without full-length and/or 3′-terminal sequences of the target RNA molecule.

Our recent findings suggest that the addition of ribonuclease H (RNase H) to the reaction mixture leads to DNA synthesis from a nick site in the RNA/DNA hybridization region^6^. Thus, the use of RNase H should permit RNA detection if the target RNA sequence is partially known. However, specific mRNA detection via RNase H-assisted RPRCA with a pre-circularized probe only works in the absence of non-target RNA molecules in the reaction mixture, presumably because pre-circularized probes can hybridize with non-target RNAs and initiate DNA synthesis upon RNA digestion by RNase H. A similar result was reported previously^7^. Therefore, the specificity of RNase H-assisted RPRCA should be improved to make RNA detection simple and practical.

Apart from RPRCA, it is well known that RCA with padlock probe technology greatly enhances the specificity for DNA detection^8,9^. The padlock probe is linear ssDNA that has a complementary sequence to target DNA molecules divided into two at both the 5′ and 3′ ends (designated as “arms” hereafter) via the linker sequence (Supplementary Fig. S1). Therefore, when both arms of the probe hybridize with the target DNA (DNA-splinted padlock probe), they are aligned on a straight via the nick, and then, the probe is circularized by sealing the nick using DNA ligase. Finally, the circularized padlock probe is used as a template for the RCA reaction.

Similarly, regarding RNA detection, two ligases, namely T4 DNA ligase (T4Dnl) and T4 RNA ligase 2 (T4Rnl2), can be used to seal an ‘RNA-splinted padlock probe’. T4Dnl can recognize an RNA-splinted padlock probe as a substrate by reducing the adenosine triphosphate (ATP) concentration in the reaction mixture to 10 µM^10–15^, although the sealing efficiency of T4Dnl is significantly lower for RNA-splinted padlock probes than for ‘DNA-splinted padlock probes’^5,16^. T4Rnl2 can also efficiently recognize an RNA-splinted padlock probe as a substrate^17–20^. However, its activity has not been confirmed by other research groups^5,16,21–23^; thus, the sealing activity of T4Rnl2 regarding RNA-splinted padlock probes remains controversial. In padlock RCA, because the detection sensitivity and specificity depend on the sealing efficiency of the padlock probe, selecting the optimal ligase is important when RNase H-assisted RPRCA is employed together with padlock probe technology.

Recently, it was demonstrated that the ATP-dependent DNA ligase of chlorella virus PBCV-1^24^, which is commercially available as SplintR ligase (New England BioLabs, Ipswich, MA, USA), can seal RNA-splinted DNA with high efficiency^15^. In this study, we developed a novel RPRCA procedure for the direct detection of mRNA molecules in combination with RNase H, a padlock DNA probe, and SplintR ligase, which can prepare a circular probe from an RNA-splinted padlock DNA probe with higher ligation efficiency than T4Rnl2 and T4Dnl. This RNase H-assisted RPRCA together with SplintR ligase provides higher specificity even in the presence of non-target RNA, suggesting that this technique represents a simple and reliable method for detecting any type of RNA molecule.

## Results

### Design of the detection scheme

**Figure 1:**
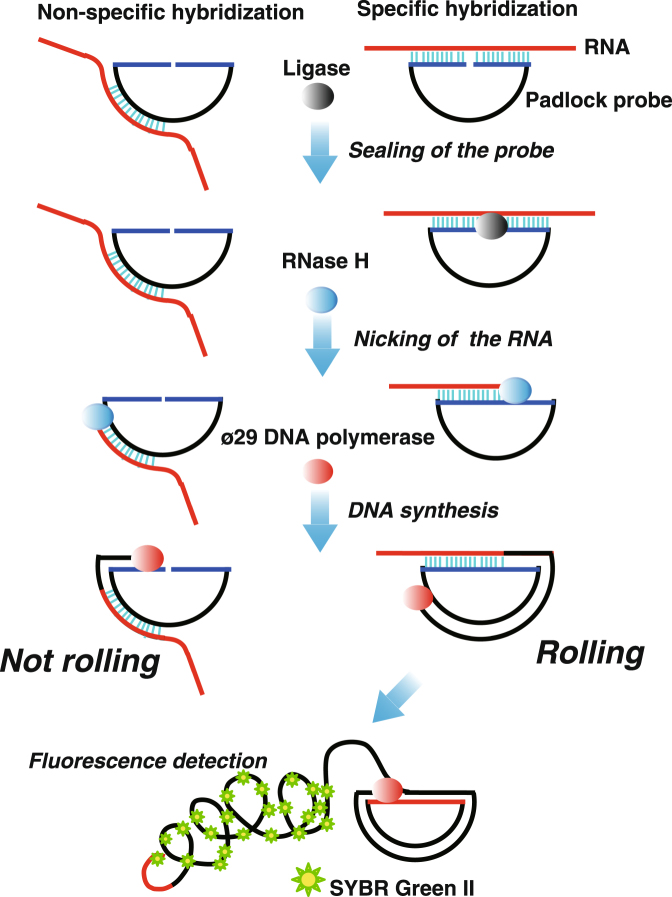
A schematic representation of targeted RNA detection by RNase H-assisted RNA-primed rolling circle amplification using a padlock probe.

Figure 1 shows a basic scheme for target RNA detection by RNase H-assisted RPRCA using a padlocked probe, which consists of five reaction steps as follows: (i) the padlock probe is hybridized to a sequence of interest in the target mRNA molecule; (ii) the hybridized padlock probe is sealed by SplintR ligase to create a circular DNA probe; (iii) RNase H produces a nick site in the hybridized mRNA; (iv) phi29 DNA polymerase catalyzes DNA synthesis at the nick site of RNA with strand displacement; and (v) finally, RPRCA synthesizes long ssDNA, which has a periodic sequence complementary to the padlock probe.

### Comparison of sealing efficiency

In our strategy, both the detection sensitivity and specificity depend on the sealing efficiency of the RNA-splinted padlock probe. Therefore, we first examined whether SplintR ligase can more efficiently seal an RNA-splinted padlock probe than T4Dnl and T4Rnl2, which were used previously^10–15,17–20^.

In this experiment, each ligase was tested under different ATP concentrations because T4Dnl could seal the RNA-splinted padlock probe only under low ATP concentrations^10–15^. CircLigase was also used to prepare circular ssDNA probes as a control for gel electrophoresis. After ligation, the ligated products were treated with multiple exonucleases to remove linear ssDNA probe remaining in the reaction mixture and successively purified and analyzed using polyacrylamide gel electrophoresis (PAGE) in the presence of 5% urea.

**Figure 2:**
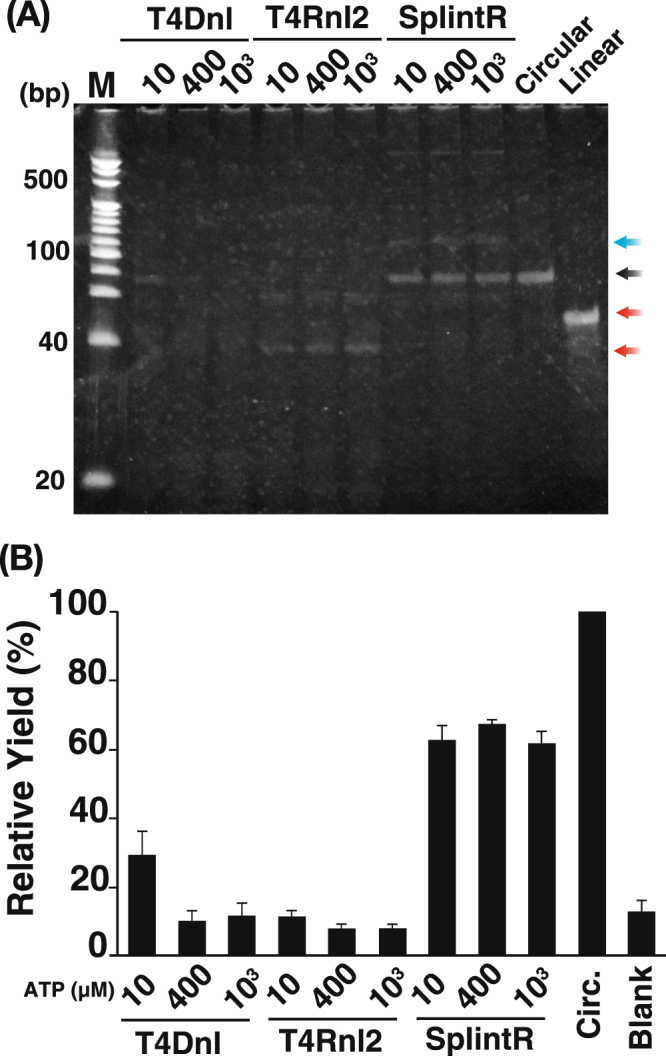
Comparison of the sealing efficiency of the RNA-splinted padlock probe under different ATP concentrations. (**A**) Denatured polyacrylamide gel analysis of products ligated by T4 DNA ligase (T4Dnl), T4 RNA ligase 2 (T4Rnl2), and SplintR ligase (SplintR) using an RNA-splinted padlock probe. The numbers above each lane indicate the ATP concentration (µM) used in the ligation reaction. M, 20-bp DNA size marker; Linear, linear padlock probe; Circular, circular padlock probe made by CircLigase. The white arrow indicates a linear padlock probe. The black arrow indicates a circularized padlock probe. The red arrows indicate oligo debris of DNase treatment. The blue arrow indicates a circularized padlock probe/RNA hybrid molecule. (**B**) Relative yield of the circularized padlock probe. The gel images were analyzed using ImageJ for comparisons with the known concentration of the pre-circularized probe made by CircLigase. The yield of each padlock probe was calculated by setting the control as 100%. Circ., circular probe made by CircLigase; Blank, blank lane. Five electrophoresis experiments were conducted using five independent reaction products, and the mean and error were calculated.

We found that irrespective of the ATP concentration, a band corresponding to the circular ssDNA probe was observable in the presence of SprintR ligase (Fig. 2A). The yield of the circularized padlock probe by SplintR ligase was stable under all ATP concentration conditions (10 µM, 62.8 ± 4.2%; 400 µM, 67.5 ± 1.1%; and 1 mM, 61.9 ± 3.4%; Fig. 2B). T4Dnl could only seal the RNA-splinted padlock probe under low ATP concentrations (10 µM) as reported previously (Fig. 2A)^10–15^. A comparison of the fluorescence intensities of the circular ssDNA probes suggested that the sealing efficiency of T4Dnl is lower than that of SplintR ligase in the presence of 400 µM ATP (Fig. 2B). However, an obvious circularized padlock probe band was often not confirmed (data not shown). As a result, the yield of the circularized padlock probe was unstable (29.3 ± 7.0%, Fig. 2B). By contrast, T4Rnl2 rarely sealed the RNA-splinted padlock probe regardless of the ATP concentration (Fig. 2A,B), contrary to previous reports^17–20^. In addition, the yield of the circularized padlock probe (10 µM, 11.53 ± 1.6%; 400 µM, 8.06 ± 1.2%; and 1 mM, 8.17 ± 1.1%; Fig. 2B) was almost identical to that of the blank lane (13.1 ± 3.2%). Indeed, an obvious circularized padlock probe band could not be confirmed by any gel electrophoresis analysis (data not shown). This experiment clearly illustrated that SplintR ligase is most suitable for the circularization of RNA-splinted padlock probes among the ligases tested.

### Evaluation of the effect of the position of the probe on sealing efficiency

In our previous study on RPRCA for mRNA detection using pre-circularized probes^6^, the probe position on GFP mRNA did not largely affect RPRCA using RNase H. This previous result indicated that the secondary structure of mRNA would not influence the ratio of mRNA hybridization with pre-circularized probes. However, RNase H-assisted RPRCA involves a ligation step with a padlock probe, and thus, the secondary structure of the probed region on the target mRNA to which the arms of the probe are hybridized might affect RPRCA. In addition, as observed previously^15^, the sealing efficiency of SplintR ligase was affected by the nucleotide pair at both arm ends of the padlock probe. Therefore, we examined whether the padlock probe position and arm sequence on the target mRNA affected the sealing efficiency of SplintR ligase.

**Figure 3:**
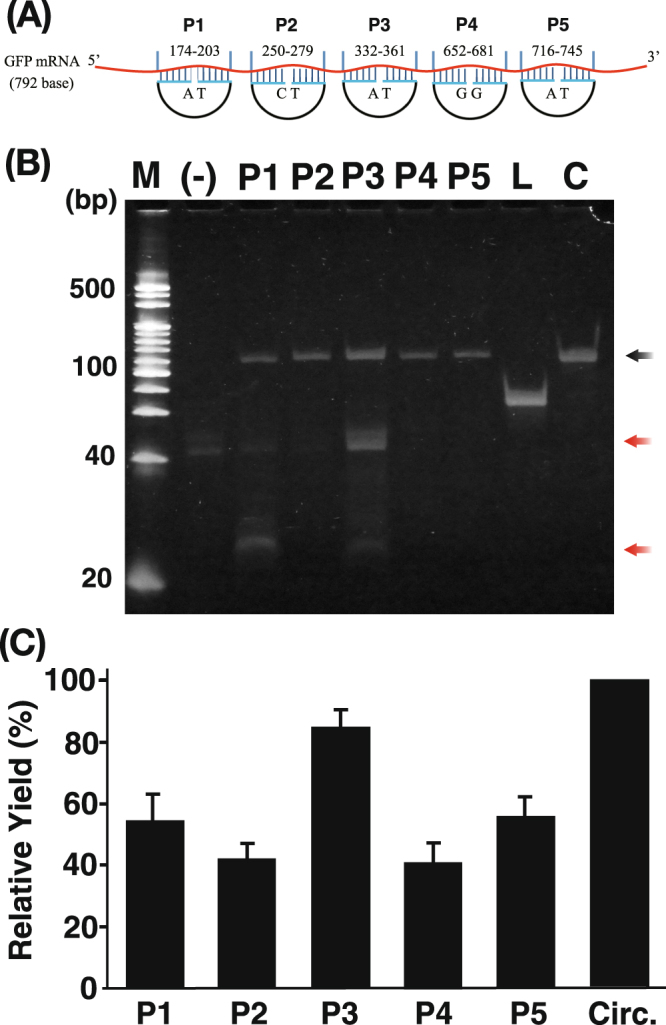
Comparison of the effect of the position of the padlock probe. (**A**) Graphical representation of each padlock probe position on *in vitro*-transcribed GFP mRNA. The numbers above each probe indicate the nucleotide numbers from the 5′ end of mRNA. The letters in each padlock probe indicate the nucleotide pair of the arm end (left, 5′ end; right, 3′ end). (**B**) Denatured polyacrylamide gel analysis of ligation products using padlock probes with different hybridizing sequences on the mRNA sequence. M, 20-bp DNA size marker. The black arrow indicates a circularized padlock probe. The red arrows indicate oligo debris of DNase treatment. L, linear probe; C, circular padlock probe made by CircLigase. (**C**) Relative yield of each circularized padlock probe. The gel images were analyzed using ImageJ for comparisons with the known concentration of pre-circularized probe made by CircLigase (Circ.) as a control. The yield of each padlock probe was calculated by setting the control as 100%. Three electrophoresis experiments were conducted with three independent reaction products, and the mean and error were calculated.

The arm positions and nucleotide pair at both arm ends of each padlock probe on the target GFP mRNA are illustrated in Fig. 3A, and the chemical characteristics of each padlock probe (e.g., the melting temperature [Tm] of the arm position) used in this study are also shown in Supplementary Table S1. GFP mRNA was prepared by *in vitro* transcription using pET-AcGFP.

We found that all padlock probes were converted into circular ssDNA by SplintR ligase (Fig. 3B,C). Although the Tm values of the arms on P1 and P3 were approximately 60 °C, the sealing efficiency was different (P1, 54.6 ± 8.1%; P3, 84.3 ± 5.9%). In addition, although the arms of P2 had higher Tm values than those of P5, the sealing efficiencies of P2 and P5 were low (P2, 41.6 ± 5.3%; P5, 55.4 ± 7.0%). These results indicate that the sealing efficiency is not simply determined by the Tm value of the arm on the padlock probe.

According to a previous report^15^, the nucleotide pair of P1, P3, and P5 (5′pdA and 3′dT) is the most efficient for sealing RNA-splinted DNA fragments. However, the sealing efficiencies of P1 (54.6 ± 8.1%) and P5 (55.4 ± 7.0%) were not significantly different from those of P2 (41.6 ± 5.3%) and P4 (40.4 ± 6.6%), which had inferior pairs (P2, 5′pdC and 3′dT; P4, 5′pdG and 3′dG) in the report. These results suggest that the sealing efficiency may be slightly affected by the secondary structure of RNA rather than the Tm and nucleotide pair of the padlock probe. In any case, this experiment clearly illustrated that the padlock probe can be set at an arbitrary position in the target RNA molecule, with some differences in efficiency.

### Determination of the optimal RNase H concentration

RNase H is generally used at higher concentrations to completely degrade RNA species on RNA/DNA hybrids; however, several nicks are required for this experiment. Therefore, in the next step, optimization of the RNase H concentration was necessary for RNase H-assisted RPRCA. After hybridization and ligation of the padlock probe by SplintR ligase, RNase H-assisted RPRCA was performed in the absence or presence of various concentrations of RNase H. Initially the resulting amplified ssDNA was analyzed by agarose gel electrophoresis.

We found that ssDNA products did not arise in the absence of both GFP mRNA and the padlock probe or in the presence of only GFP mRNA or the padlock probe. Rather, the addition of RNase H to the samples promoted the RPRCA reaction (Supplementary Fig. S2). ssDNA synthesized by RPRCA was apparently saturated in the presence of 0.03 units or more of RNase H; however, it was difficult to compare the small yields of synthesized DNA in the confirmation of gel electrophoresis.

**Figure 4:**
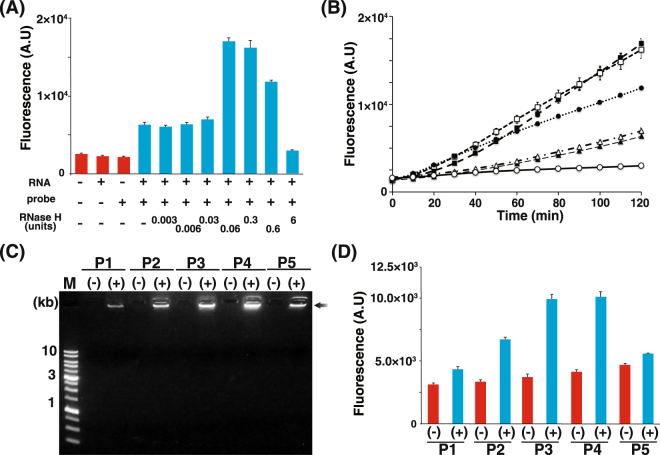
Quantification and real-time monitoring of RNase H-assisted RNA-primed rolling circle amplification (RPRCA) products. (**A**) Quantification of the amplification products generated using various concentrations of RNase H with padlock probe P3 in real time. (**B**) Real-time monitoring of RNase H-assisted RPRCA products generated using various concentrations of RNase H with padlock probe P3. The amounts of RNase H used were as follows: open circles, 6 units; closed circles, 0.6 units; open squares, 0.3 units; closed squares, 0.06 units; open triangles, 0.03 units; and closed triangles, 0.006 units. The data of 0.003 units and all negative controls were omitted. (**C**) Agarose gel analysis to compare the effect of the position of padlock probes (250 fmol) in an RNase H-assisted RPRCA reaction. All reactions used 10 fmol of *in vitro*-transcribed mRNA. M, 1-kb ladder DNA size marker; + , presence of RNase H (0.06 units); −, absence of RNase H. The arrow indicates an RCA product. (**D**) Quantification of the effect of the position of the padlock probes (250 fmol) in an RNase H-assisted RPRCA reaction using a real-time system.

Therefore, to clarify the optimal RNase H amount, we measured the yield of synthesized ssDNA with SYBR Green II in real time for 2 h. According to the final yield, 0.06–0.3 units of RNase H would be suitable for RNase H-assisted RPRCA for the specific detection of GFP mRNA (Fig. 4A). In addition, DNA was not amplified when RNase H was excessively used in the reaction (e.g., 6 units per reaction), presumably because all of the RNA in the RNA/DNA hybridized region was degraded. Conversely, according to real-time monitoring, the amount of synthesized DNA is constant using 0.6 units of RNase H, whereas the amount is slightly increased with time when using 0.06–0.3 units of RNase H (Fig. 4B). This result indicates that the initiation of DNA synthesis was performed several times by using optimal amounts of RNase H.

Then, we examined whether the padlock probe position affected the detection sensitivity using five probes with or without RNase H via agarose gel electrophoresis. DNA synthesis was detected using all probes only when RNase H was present (Fig. 4C). Furthermore, background DNA synthesis was not observed in the absence of RNase H, even if the reaction time was extended to 12 h (data not shown). However, the final yield of ssDNA differed for each probe, indicating that the detection sensitivity depended on the probe position. To investigate the effect of the probe position, we measured the yield of synthesized ssDNA with SYBR Green II in real time for 2 h. According to the final yield, the detection efficiencies of P1 and P5 were inferior to those of P2 and P4 even though the sealing efficiencies of the probes were similar (Fig. 4D). By contrast, the detection efficiency of P4 was similar to that of P3 even though the sealing efficiency of the P4 probe was inferior. This result suggests that the detection efficiency of RNase H-assisted RPRCA might be affected by factors other than the sealing efficiency of the padlock probe.

### Specific detection of GFP mRNA

**Figure 5:**
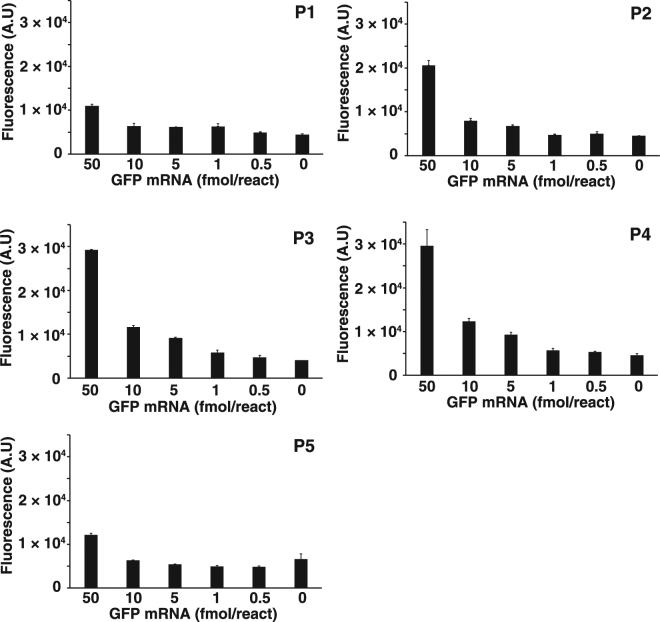
Comparison of the detection limit of each probe using *in vitro*-transcribed GFP mRNA spiked into a known yield of total RNA (60 ng) from *E. coli* (strain BL21) in real time. RNase H was used at 0.06 units per reaction. All reactions were performed in triplicate, and the mean and error were calculated.

In our preliminary study, RPRCA using a pre-circularized probe combined with RNase H could not distinguish the target GFP mRNA from other RNA because RNase H also non-specifically converted hybridized RNA into a primer for RCA. Thus, to determine the influence of non-target RNA molecules, RNase H-assisted RPRCA was performed with *in vitro*-transcribed GFP mRNA spiked into a constant amount of *E. coli* total RNA (60 ng) consisting mainly of ribosomal RNA (rRNA) and transfer RNA (tRNA). In addition, to investigate the sensitivity of each probe, we measured the yield of synthesized ssDNA with SYBR Green II in real time for 2 h. According to the real-time monitoring, the detection sensitivity was at least 5 fmol for the higher-sensitivity probes (P2, P3, and P4) and at least 10 fmol for the lower-sensitivity probes (P1 and P5) (Fig. 5). Furthermore, total RNA from *E. coli* cells was not synthesized into DNA using any padlock probe. Thus, RNase H-assisted RPRCA could clearly distinguish GFP mRNA from other RNA species such as rRNA and tRNA that constituted the majority of total RNA.

### Direct detection of GFP mRNA in total RNA from *E. coli* cells

**Figure 6:**
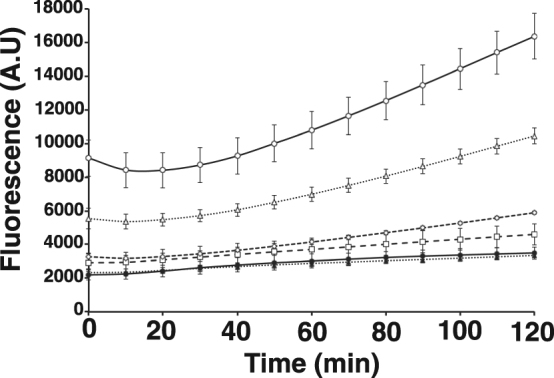
Real-time detection of GFP mRNA present in total RNA isolated from *E. coli* carried pET-AcGFP with induction. The amounts of total RNA used were as follows: open circles, 80 ng; open triangles, 40 ng; open diamonds, 20 ng; open squares, 10 ng; closed circles, 2 ng; and closed triangles, 0 ng. RNase H was used at 0.03 units per reaction. All reactions were performed in triplicate, and the mean and error were calculated.

Finally, we used serially diluted total RNA to assess the detection sensitivity of RNase H-assisted RPRCA by real-time monitoring using P4, which was one of the most sensitive probes. Total RNA was extracted from *E. coli* cells carrying the pET-AcGFP vector with or without the induction of GFP gene expression. We found that GFP mRNA could be detected from at least 10 ng of total RNA within 2 h (Fig. 6). This detection sensitivity of RNase H-assisted RPRCA using P4 was virtually identical to that of conventional RPRCA using pre-circularized probes^3^.

## Discussion

In the present study, we developed a simple method for the direct detection of sequences of interest in target RNA molecules without RT. RNase H specifically recognized the target RNA hybridized with a circularized ssDNA probe, which was produced using an RNA-splinted padlock probe and SplintR ligase.

Our first concern in this study was that the signal-to-noise ratio worsened due to background DNA synthesis from non-circularized padlock probes, as we reported previously^3,25^. SYBR Green II non-specifically stains both ssDNA and double-stranded DNA. Therefore, the fluorescence of SYBR Green II does not necessarily reflect ssDNA synthesized via the RCA reaction. In addition, unlike PCR, the product length in RCA cannot be used to evaluate whether correct DNA synthesis is occurring. Awkwardly, based on the result of gel electrophoresis, this background DNA is probably long ssDNA, and it often contains homologous sequences to fluorescence-labeled detection probes because this background DNA might be generated using the padlock probe as a template. Therefore, we believe that the elimination of background DNA synthesis is important for securing the reproducibility and specificity in our detection system as well as the entire RCA reaction.

Unexpectedly, no background DNA synthesis was detected in this study. The main reason is that our strategy, rolling circle DNA synthesis, does not start unless the formation of a primer after circularization of the padlock probe by RNase H is functioning accurately. Conversely, we believed two other factors eliminated background DNA synthesis, namely DNA-free phi29 DNA polymerase and the use of a bench-top extra cleanroom classified as international standard ISO-1 according to ISO 14644-1^26^ set by the International Organization for Standardization (Geneva, Switzerland). DNA-free phi29 DNA polymerase can reduce contaminating DNA including ssDNA^27^. Consequently, the molecular numbers of the 3′ end of ssDNA were significantly reduced in the reaction compared with the findings in previous experiments. For this reason, we believe that DNA-free phi29 DNA polymerase is an important component to secure the reproducibility and specificity of the RCA reaction. A bench-top extra cleanroom classified as ISO-1 featuring an ionizer to eliminate static charges from plastic ware^28^ was used to extract total RNA and prepare all reaction mixtures including those for *in vitro* transcription. Airborne particles, which cannot be recognized by the naked eye, affect DNA amplification^28,29^, and the ionizer lessened accidental contamination regardless of the operator’s experience. Using these two special tools, background DNA synthesis was suppressed in both whole genome amplification (WGA) and RCA reactions.

Interestingly, both the detection sensitivity and specificity of our strategy depended on the sealing efficiency of the RNA-splinted padlock probe. Therefore, selecting the optimal ligase was important for our strategy. T4Dnl could seal the RNA-splinted padlock probe only in the presence of low ATP concentrations^10–15^. T4Rnl2 has been routinely used to ligate DNA oligonucleotides to the 3′ end of RNA^24,30^. However, it is unknown whether T4Rnl2 can seal RNA-splinted padlock probes. Therefore, we sought to determine which ligase was most efficient for sealing RNA-splinted padlock probes. Our results clearly illustrated that T4Rnl2 had little ability to join nicked DNA strands even when the DNA annealed to RNA. By contrast, T4Dnl and SplintR ligase could seal RNA-splinted padlock probes, and SplintR ligase was more suitable for this process. Therefore, we speculated that the results of previous reports using T4Rnl2^17–20^ for RPRCA reflected background DNA synthesis from non-circularized padlock probes or contaminating DNA in the reaction, which frequently occurs in both RCA and WGA using phi29 DNA polymerase^3,25,31^. Incidentally, we could not perform the ligation experiment in the presence of 400–500 mM ATP as described previously^18,19^ because ATP was not dissolved in the buffer at these concentrations.

Meanwhile, previous reports described that phi29 DNA polymerase degraded RNA from the 3′ end to the RNA/DNA hybridized region with 3′ to 5′ exoribonuclease activity (or in combination with RNase III to remove the double-stranded RNA region), permitting phi29 DNA polymerase to transform the target RNA into a primer^32,33^. However, the 3′ to 5′ exoribonuclease activity of phi29 DNA polymerase has not been confirmed by our team or other researchers^34^. Furthermore, our results revealed that RNase H was more effective for RNA sequence detection in RPRCA even though phi29 DNA polymerase has 3′ to 5′ exoribonuclease activity.

Two recent papers^35,36^ reported that *in situ* RCA can detect eukaryotic mRNA using a padlock probe and SplintR ligase. Previously, RCA for detecting eukaryotic mRNA was performed after RT using target-specific primers^34^. Therefore, this method combining a padlock probe with SplintR ligase enabled the RCA reaction for *in situ* mRNA detection without RT. Thus, RNase H-assisted RPRCA would be also suitable for *in situ* mRNA detection even if the RNA has a poly(A) tail.

However, different from our method, this previous method employed an extra primer to recognize the padlock sequence for RCA as a detection primer. Moreover, the previous methods do not allow setting of the padlock probe at an arbitrary region in the target RNA molecule. Because SplintR ligase can also seal a DNA-splinted padlock probe, the detection primer could amplify the circularized padlock probe generated via hybridization with genomic DNA. To prevent DNA synthesis from the DNA-splinted padlock probe in their method, the padlock probe was set at an exon-exon junction on the mRNA molecule to detect only RNA. Thus, the method is not suitable for detecting single-exon mRNAs (e.g., most mRNAs of *Saccharomyces cerevisiae*). RNase H cannot convert “DNA-splint” into a primer; therefore, our procedure using RNase H can detect only target RNA molecules even if SplintR ligase can also seal the DNA-splinted padlock probe.

Contrarily, this RNase H-assisted RPRCA did not have better detection sensitivity than the previous technique using the 3′ end of RNA as a primer. We believe that the detection sensitivity of both RPRCA strategies depends on the sensitivity of SYBR Green II (1 ng/reaction). By our calculations, the detection limit of RPRCA using SYBR Green II is nearly 1 × 10^7^ copies (Supplementary Text 1). Therefore, further improvement of the detection limit cannot be expected unless the detection method is changed.

In summary, our procedure has the potential to detect almost all types of RNA molecules without RT and full-length sequence information, especially for 3′-terminal sequences.

## Materials and Methods

### Solutions, mixtures, plastic ware, and DNA

To avoid contamination from the laboratory environment, prepared solutions supplied by the manufacturers were used as much as possible in this study. UltraPURE™ distilled water and Tris-HCl solution (pH 7.5) were purchased from Thermo Fisher Scientific (Grand Island, NY, USA). All homemade solutions and buffers were filtrated and sterilized using a 0.1-µm-pore-size polyethersulfone membrane bottle top filter unit (Nalgene/Thermo Fisher Scientific, Rochester, NY, USA). Similarly, disposable sterile plastic ware was used whenever possible to reduce the likelihood of DNA and RNase contamination. Aerosol-resistant filter tips were purchased from Molecular BioProducts/Thermo Fisher Scientific (San Diego, CA, USA), and microcentrifuge tubes and PCR-grade 0.2-ml tubes were obtained from Eppendorf (Hamburg, Germany). The 1-kb DNA ladder marker was acquired from Maestrogen (Las Vegas, NV, USA). The 20-bp ladder marker was purchased from Takara Bio (Otsu, Shiga, Japan). All solutions and mixtures were prepared using dedicated sets of pipettes in an exclusive bench-top cleanroom (KOACH 500 F, Koken Ltd, Tokyo, Japan) that was classified as ISO-1 after cleaning using RNase *AWAY* (Molecular BioProducts, San Diego, CA, USA).

### Padlock probe design

Unlike PCR primer design, padlock probe design does not have a fixed rule. In many cases, the probe length is less than 100 nucleotides, and the length of the arm is less than 20 nucleotides. As the process basically involves the hybridization of nucleotides, the Tm of the arm should be higher than the reaction temperature in the ligation step. The Tm of the arm was calculated using OligoAnalyzer 3.1 (http://sg.idtdna.com/calc/analyzer) from Integrated DNA Technologies under the following conditions: oligo concentration, 10 µM; Na concentration, 50 mM; Mg concentration, 10 mM; and target, RNA. In our preliminary experiments, the linker should be longer than the sum of the arm sequences. We believe this is probably due to the physical effect of the rigidity of ssDNA. In our preliminary experiments, the secondary structure of the probe significantly affected the ligation efficiency, especially when the arm region hybridized with the linker region (data not shown). Therefore, we evaluated the secondary structure of the probe using OligoAnalyzer 3.1 at the probe design stage. All padlock probes were designed on the basis of these findings.

### Oligonucleotides

The ribonucleotide for testing ligation efficiency was purchased from Japan Bio Services Co., LTD (Saitama, Japan). All padlock probes were purchased from Eurofins Genomics, Inc. (Tokyo, Japan). All oligomers were dissolved in 0.1x TE buffer (1 mM Tris-HCl pH 7.5, 0.01 mM EDTA) in the cleanroom as described previously. The sequences and Tm values of padlock probes for detecting GFP mRNA are shown in Supplementary Table 1.

### Comparison of circularization efficiency of RNA-splinted padlock probes

To prepare the hybridization mixture, 25 pmol of the ribonucleotide (5′-rGrCrGrArUrCrArCrArUrGrArUrCrUrArCrurUrCrGrGrCrUrUrCrGrUrGrA-3′, 30-mer) and 100 pmol of the padlock probe for testing ligation efficiency (5′Pho-AGATCATGTGATCGCgaattcgccagggttttcccagtcacgactTCACGAAGCCGAAGT-3′, 60-mer, uppercase letters are homologous to the ribonucleotide sequence) were added to a mixture containing 20 mM Tris-acetate (pH 7.5), 50 mM potassium glutamate (KGlu), and 0.1 mM EDTA in a final volume of 10 µl. Then, the hybridization mixture was incubated at 95 °C for 1 min followed by immediate cooling to 40 °C and incubation for 10 min at 30 °C. After hybridization, 10 µl of a ligase mixture containing 20 mM Tris-acetate (pH 7.5), 20 mM magnesium acetate (MgAc), 50 mM KGlu, 20, 800, or 2000 µM ATP, and each ligase (200 units of T4Dnl, 5 units of T4Rnl2, and 12.5 units of SplintR ligase, New England BioLabs) were added to the hybridization mixture and then incubated at 37 °C for 1 h to seal the padlock probe, followed by enzyme inactivation at 65 °C for 10 min. After ligation, 10 µl of a nuclease mixture containing 20 mM Tris-acetate (pH 7.5), 20 mM MgAc, 50 mM KGlu, 1 mM ATP, 10 units of Plasmid-Safe DNase (Epicentre Technologies, Madison, WI, USA), 10 units of exonuclease III (Takara Bio), and 10 units of exonuclease I (New England BioLabs) were added to the ligation mixture, which was then incubated at 37 °C for 15 min to degrade linear single-stranded DNA (ssDNA). After enzyme inactivation at 80 °C for 15 min, the circular ssDNA was purified using a High Pure PCR Cleanup Micro Kit (Roche Diagnostics GmbH, Mannheim, Germany). The purified DNA was analyzed using 10% PAGE containing 7 M urea (Tris-Borate-EDTA buffer) and visualized by staining using UltraPower™ DNA/RNA Safedye (Gellex International, Tokyo, Japan) and a blue-light transilluminator. The gel images were analyzed using NIH Image software (ImageJ) for comparisons with the known concentration of the pre-circularized probe made by CircLigase. The yield of each padlock probe was calculated by setting the control as 100%. These ligation reactions and electrophoresis were both performed five times.

### *In vitro* transcription of GFP mRNA

*In vitro* transcription of mRNA was performed using a HiScribe™ T7 High Yield RNA Synthesis Kit (New England BioLabs) according to the manufacturer’s protocol. After linearization via *Not*I (Takara Bio) digestion, 1 µg of pET-AcGFP^3^ was added to the transcription reaction mixture, which was incubated at 37 °C for 2 h. After RNase-free DNase I (Takara Bio) treatment to remove the template DNA, the transcribed mRNA was then purified using a NucleoSpin® RNA Clean-up XS kit (Macherey-Nagel, Düren, Germany) according to the manufacturer’s protocol, and RNA concentrations were determined using a Qubit® fluorometer (Invitrogen/Thermo Fisher Scientific, Waltham, MA, USA) with Qubit® RNA BR Assay Kits (Invitrogen). After denaturation with formamide, RNA quality was checked via agarose gel electrophoresis (1.5%, Tris-acetate EDTA [TAE] buffer) and visualized by staining using UltraPower™ DNA/RNA Safedye and a blue-light transilluminator. The transcribed mRNA was stored at −80 °C until required.

### Comparison of circularization efficiency according to the padlock probe position

To prepare the hybridization mixture, 25 pmol of the *in vitro* transcribed GFP mRNA and 100 pmol of the each padlock probe (P1, P2, P3, P4, and P5, Supplementary Table 1) were added to a mixture containing 20 mM Tris-acetate (pH 7.5), 50 mM KGlu, and 0.1 mM EDTA in a final volume of 10 µl. Then, the hybridization mixture was incubated at 95 °C for 1 min followed by immediate cooling to 40 °C and incubation for 10 min at 30 °C. After hybridization, 10 µl of a ligase mixture containing 20 mM Tris-acetate (pH 7.5), 20 mM MgAc, 50 mM KGlu, 20, 800, or 2000 µM ATP, and 12.5 units of SplintR ligase were added to the hybridization mixture and then incubated at 37 °C for 1 h to seal the padlock probe, followed by enzyme inactivation at 65 °C for 10 min. After ligation, 10 µl of a nuclease mixture containing 20 mM Tris-acetate (pH 7.5), 20 mM MgAc, 50 mM KGlu, 1 mM ATP, 10 µg of RNase (Nacalai Tesque, Kyoto, Japan), 10 units of Plasmid-Safe DNase, 10 units of exonuclease III, and 10 units of exonuclease I were added to the ligation mixture, which was incubated at 37 °C for 15 min to degrade linear single-stranded DNA (ssDNA). After enzyme inactivation at 80 °C for 15 min, the circular ssDNA was purified using a High Pure PCR Cleanup Micro Kit. After denaturation using urea, the purified DNA was analyzed using 10% PAGE containing 7 M urea (Tris-Borate-EDTA buffer) and visualized by staining using UltraPower™ DNA/RNA Safedye and a blue-light transilluminator. The gel images were analyzed using ImageJ for comparisons with the known concentration of the pre-circularized probe made by CircLigase as a control. The yield of each padlock probe was calculated by setting the control as 100%. These ligation reactions and electrophoresis were both performed three times.

### Expression of GFP mRNA in *E. coli* and total RNA extraction

The *E. coli* strain BL21 (DE3) (Novagen, Darmstadt, Germany) was transformed with pET-AcGFP^3^ and then grown on an LB agar plate (1% tryptone, 0.5% yeast extract, 1.0% NaCl, and 1.5% agar) containing 50 µg/ml ampicillin to select clones transformed with pET-AcGFP. These cells were then grown in LB medium for 8 h at 30 °C. The cultures were diluted 1:1000 in fresh LB medium with or without the Overnight Express™ Autoinduction System (Novagen) to induce GFP mRNA transcription and incubated at 30 °C with shaking for 12 h. The *E. coli* strain BL21, which has no vector, was also cultured at 30 °C for 8 h with LB medium. Cells were harvested in a 50-ml conical tube by centrifugation at 2150 × *g* for 20 min at 4 °C. Total RNA was isolated using a Cica Geneus Total RNA Prep kit (for tissue, Kanto Chemical, Tokyo, Japan) according to the manufacturer’s protocol. Total RNA concentrations were determined using a Qubit® fluorometer with Qubit® RNA BR Assay Kits. After denaturation using formamide, a small amount of genomic DNA contamination and degradation of RNA were confirmed in each sample via agarose gel electrophoresis (1.5%, TAE buffer) and visualized by staining using UltraPower™ DNA/RNA Safedye and a blue-light transilluminator.

### RNase H-assisted RPRCA

Either *in vitro*-transcribed GFP mRNA or total RNA (appropriate concentration, Figs 4–6) was mixed with 250 fmol of a padlock probe in a buffer containing 20 mM Tris-acetate (pH 7.5), 10 mM MgAc, 1 mM ATP, and 50 mM KGlu in a final volume of 9 µl. Hybridization of the padlock probe was facilitated by incubation at 95 °C for 1 min followed by immediate cooling to 40 °C and incubation for 10 min at 30 °C. After hybridization, 1 µl of SplintR ligase (25 units) was added to the reaction mixture, which was incubated at 37 °C for 15 min to seal the padlock probe. The RNase H-assisted RPRCA reaction was started by mixing 10 µl of the ligated mixture with 10 µl of a reaction mixture containing 20 mM Tris-acetate (pH 7.5), 10 mM MgAc, 80 mM ammonium sulfate ((NH_4_)_2_SO_4_), 10 mM KGlu, 2.0 mM deoxynucleoside triphosphate, 10 mM dithiothreitol, 0.002 units of pyrophosphatase (New England BioLabs), an appropriate number of units of RNase H (Figs 4–6, BioAcademia, Ibaraki, Osaka, Japan), and 100 ng of DNA-free phi29 DNA polymerase (Kanto Chemical). Following incubation at 30 °C for 2 h, 15 µl of the synthesized DNA were then analyzed using agarose gel (1.0%, TAE buffer) electrophoresis and visualized by staining using UltraPower DNA/RNA Safedye and a blue-light transilluminator. All reactions and electrophoresis were repeated at least three times, and representative gel electrophoresis results are shown in all figures.

For real-time detection, RNase H-assisted RPRCA was performed using a 1× concentration of SYBR Green II (Invitrogen) in a 96-well PCR plate, and fluorescence signals were measured every 10 min for 2 h with the FAM filter (excitation wavelength: 482 nm, fluorescence wavelength: 536 nm) of the Thermal Cycler Dice Real-Time System II (TP900, Takara Bio). All real-time RNase H-assisted RPRCAs were performed in triplicate to estimate the experimental variance. After real-time detection, the PCR plate was additionally incubated for 2 h followed by enzyme inactivation at 65 °C for 10 min, and the products in each well were visualized using a blue-light transilluminator to confirm the fluorescence signals.

## Electronic supplementary material

Supplementary information and text
