# Supplementary material for: RNase H-assisted RNA-primed rolling circle amplification for targeted RNA sequence detection
Source: Sci Rep. 2018 May 17;8:7770. doi: 10.1038/s41598-018-26132-x (PMC5958062; doi:10.1038/s41598-018-26132-x)
Supplement: Supplementary file 1 — Supplementary information and text [file 41598_2018_26132_MOESM1_ESM.pdf]

RNase H-assisted RNA-primed rolling circle amplification for targeted RNA sequence detection

Hirokazu Takahashi[1, 2,*], Masahiko Ohkawachi[1], Kyohei Horio[1], Toshiro Kobori[3], Tsunehiro Aki[1, 2], Yukihiko Matsumura[2, 4], Yutaka Nakashimada[1, 2] & Yoshiko Okamura[1, 2,*]

[1] *Graduate School of Advanced Sciences of Matter, Hiroshima University, Higashihiroshima, Hiroshima, 739-8530, Japan*

[2] *Core Research for Evolutional Science and Technology (CREST), Japan Science and Technology Agency (JST), Sanbancho 5, Chiyoda-ku, Tokyo 102-0075, Japan*

[3] *Division of Food Biotechnology, Food Research Institute, National Agriculture and Food Research Organization, Tsukuba, Ibaraki, 305-8642, Japan*

[4] *Division of Energy and Environmental Engineering, Hiroshima University, Higashi-Hiroshima, Hiroshima, 739-8527, Japan*

To whom correspondence should be addressed: Yoshiko Okamura, Email: okamuray@hiroshima-u.ac.jp

Supplementary information

Name	Sequence	Length	Left arm Tm[1]	Right arm Tm[1]
P1	5'-AGGTGGCATCGCCCTttccttttacgacctcaatgctgctgctgtactactcttcGGGTCAGCTTGCCGT-3'	70 mer	64.7°C	65.3°C
P2	5'-CAGGGTGGTCACCAGttccttttacgacctcaatgctgctgctgtactactcttcCTGCACGCCGTAGCT-3'	70 mer	58.8°C	59.5°C
P3	5'-AGCCCTCAGGCATGGttccttttacgacctcaatgctgctgctgtactactcttcTGCGCTCCTGGATGT-3'	70 mer	62.6°C	58.6°C
P4	5'-GTTATCGGGCAGCAGttccttttacgacctcaatgagatctgtgtactactcttcGGTGGACAGGTAGTG-3'	70 mer	54.7°C	53.6°C
P5	5'-AGATCATGTGATCGCttccttttacgacctcaatgctgctgctgtactactcttcTCACGAAGCCGAAGT-3'	70 mer	45.4°C	45.6°C

Supplementary Table S1. Sequence and data of padlock probe for detection of GFP mRNA. Uppercase letters indicate homologous sequence to GFP mRNA .

[1], Tm was caliculated by OligoAnlyzer 3.1[2] under following condition: Oligo conc., 10 µM; Na conc., 50 mM; Mg conc., 10 mM; Target, RNA.

[2], http://sg.idtdna.com/calc/analyzer

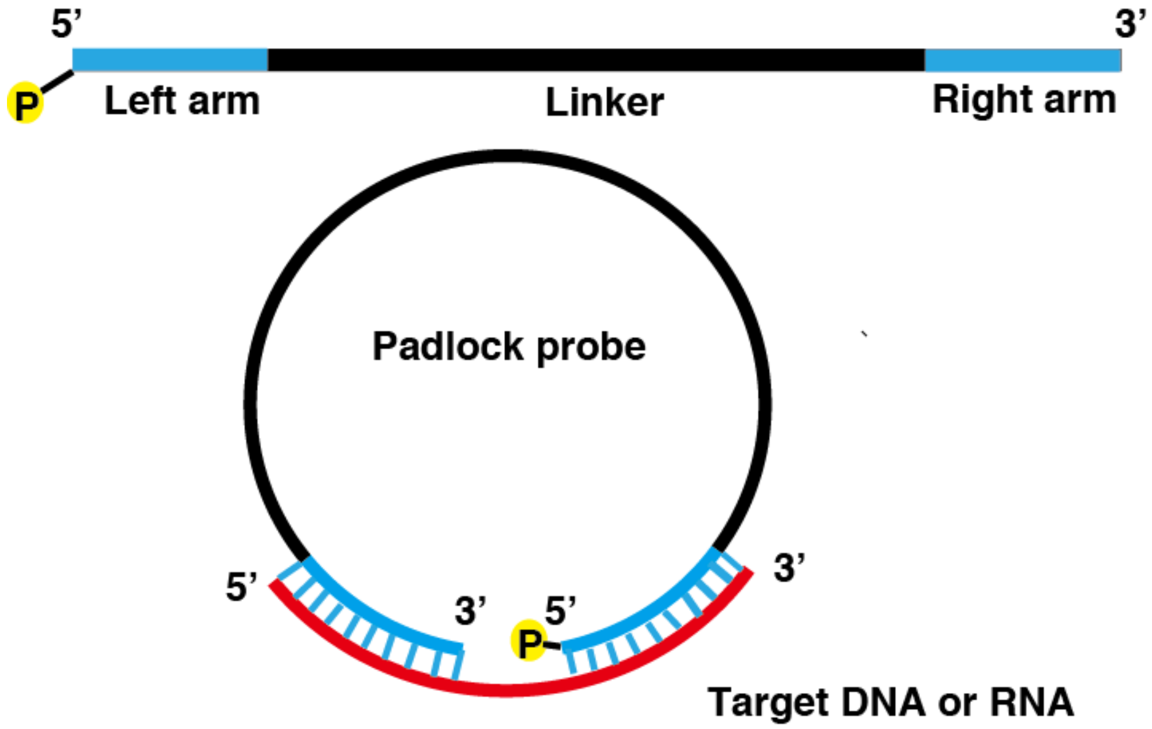

Supplementary Figure S1. Conceptual diagram of a padlock probe.

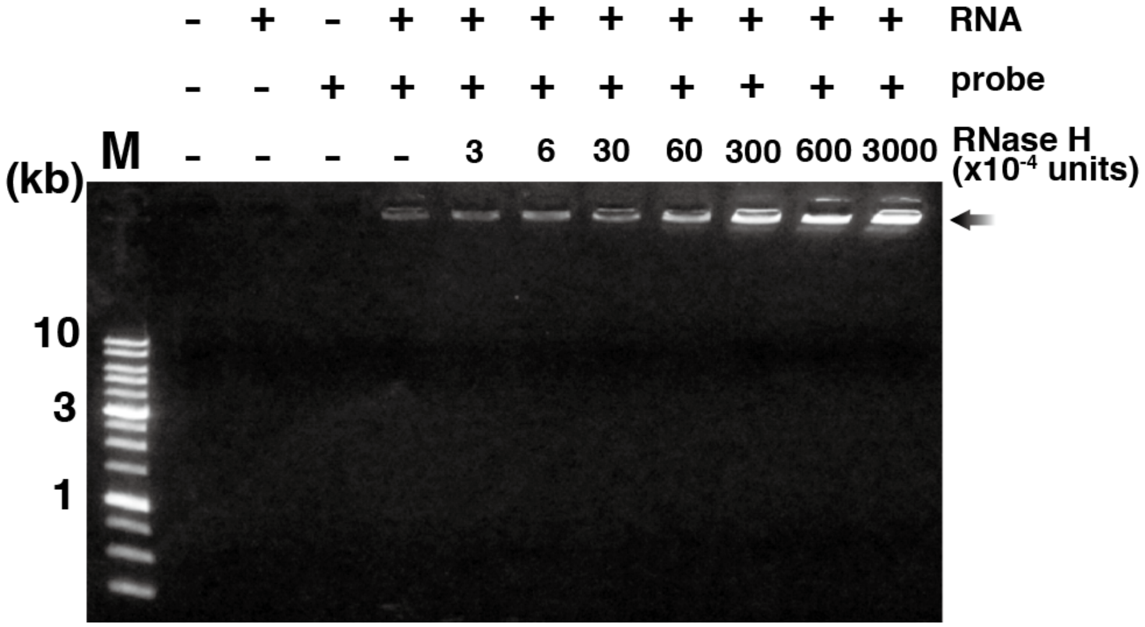

Supplementary Figure S2. Comparison of amplification products generated using various concentrations of RNase H with padlock probe P3. Numbers on the lane indicate the amount of RNase H used in the reaction. M, 1-kb ladder DNA size marker; +, presence of the probe (250 fmol) or *in vitro*-transcribed mRNA (10 fmol); −, absence of the probe, mRNA, or RNase H. The arrow indicates an RCA product.

Supplementary Text

As the DNA synthesis rate (*Ds*) of phi29 DNA polymerase is 50–200 bases/s (from Lasken 2005)[1], the maximum length (*S(l)*) of the DNA synthesized by the 2-h reaction is

$$S(l) = 200\ (Ds) \times 60\ (s) \times 120\ (m) = 1{,}440{,}000\ (bases).$$

The weight (*D (w)*) of 1 mol (6.02×10^{24} copies) of 1,440,000 (bases) is

$$D(w) = 1{,}440{,}000 \times 330 = 475{,}200{,}000\ (g).$$

Thus, the yield of ssDNA (*R(y)*) that can be generated from one copy of the probe by RCA is

$$6.02 \times 10^{24}\ copies = 475{,}200{,}000\ (g)$$
$$6.02 \times 10^{18}\ copies = 475.2\ (g)$$
$$6.02 \times 10^{15} copies = 475.2\ (mg)$$
$$6.02 \times 10^{12} copies = 475.2\ (\mu g)$$
$$6.02 \times 10^{9} copies = 475.2\ (ng)$$
$$6.02 \times 10^{6} copies = 475.2\ (pg)$$
$$6.02 \times 10^{3} copies = 475.2\ (fg)$$
$$6.02\ copies = 475.2\ (ag).$$

$$\text{Thus, } R(y) = 1\ copy \approx 78.9369\ (ag).$$

Conversely, because the minimum yield (*Min(y)*) of quantification using SYBR Green II is 1 ng according to Invitrogen, the quantitation limit (*Dl*) is calculated using the following equation:

$$Dl = \frac{Min\,(y)}{R\,(y)} = \frac{1\,(ng)}{78.9369\,(ag)} = \frac{1 \times 10^{3}(pg)}{78.9369\,(ag)} = \frac{1 \times 10^{6}(fg)}{78.9369\,(ag)} = \frac{1 \times 10^{9}(ag)}{78.9369\,(ag)}$$

$$\approx 12{,}668{,}346.489\ copies \approx 1.3 \times 10^{7} copies.$$

From this calculation, the detection limit of RCA using SYBR Green II is nearly 1 × 10^7 copies.

1 Lasken, R. S. *Multiple displacement amplification of genomic DNA*. 99-118 (Scion Publishing Ltd: UK, 2005).
